# Supplementary material for: Alpha-Germanium Nanolayers for High-Performance Li-ion Batteries
Source: Nanomaterials (Basel). 2022 Oct 26;12(21):3760. doi: 10.3390/nano12213760 (PMC9655185; doi:10.3390/nano12213760)
Supplement: Supplementary file 1 [file nanomaterials-12-03760-s001.zip › nanomaterials-1991521-supplementary.pdf]

Supplementary Materials

Section S1: Methods

Powder X-ray diffraction (PXRD) measurements were carried out on a Bruker D8 with CuKα radiation with a rapid detector (lynxeye).

Raman spectra were recorded with a BWS415 i-Raman coupled to a BAC151B microscope equipped with 20x and 50x objectives and a 532 nm laser as excitation light. All the spectra were recorded with the 50x objective, laser powers from 0.7 to 35 mW and integration times between 5000 and 20000 ms. We mapped different regions of the sample to confirm the reliability of the measured spectra. Finally, we have checked the laser power effect to determine if the presence of Ge oxides is due to our battery tests or their formation is induced by the laser.

Transmission Electron Microscopy (TEM) images were recorded in a JEOL JEM 3000F TEM system with an accelerating voltage of 300 kV.

SEM images were obtained on a Philips XL 30 S-FEG microscope operating at an accelerating voltage of 10 kV.

XPS core level spectra were obtained using Mg Kα (1253.6 eV) and Al Kα (1486.6 eV) and a Phoibos 150 electron analyzer whose axis coincided with the surface normal. The core level binding energies are calibrated using the binding energies of C 1s and Au 4f in contact with the sample as references. The line shape of core levels is fitted using a Shirley background and asymmetric singlet pseudo-Voigt functions. The fit is optimized using a Levenberg-Marquardt algorithm with a routine running in IGOR Pro (WaveMatrix Inc.) [1]The quality of the fit is judged from a reliability factor, the normalized χ^2.

The electrochemical measurement was performed using Biologic multichannel potentiostatic-galvanostatic system with impedance module BSC-815. Cyclic voltammetry was evaluated for all meameasurementstween 1.5 and 0.02 V at a scan rate of 0.1 $mV{\cdot}s^{-1}$. Galvanostatic charge-discharge (GCD) was performed between 1.8 to 0.02 V Electrochemical impedance spectroscopy measurements were carried out in a frequency range between 10kHz and 0.01Hz with an amplitude of 10 mV at open circuit potential (OCP). For analyzing the EIS we simulated with a Zview® software from Scribner.

Section S2: Physico-chemical characterization

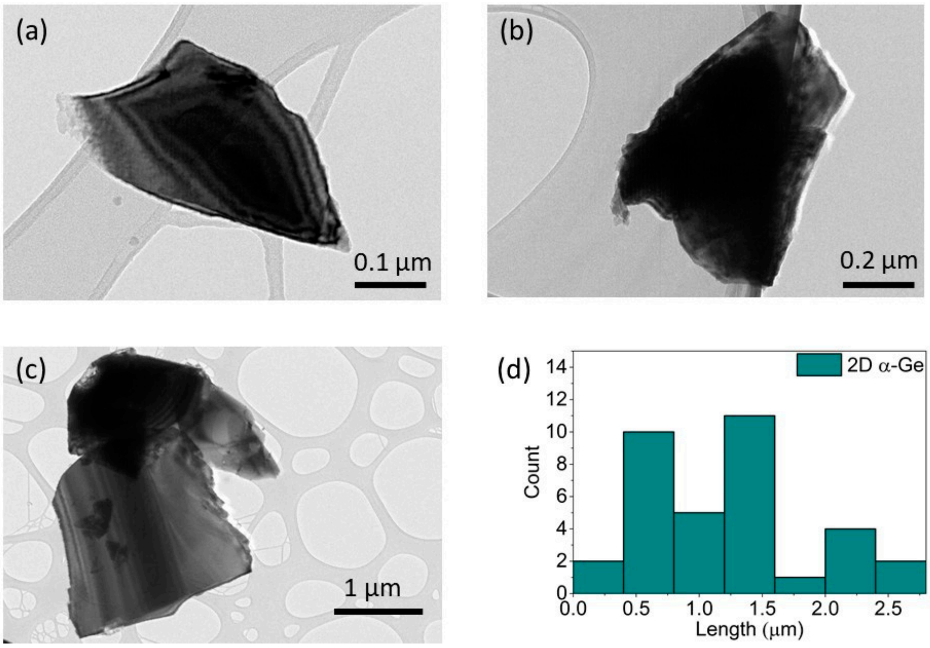

Figure S1. TEM images of 2D α-Ge (a-c) and particle size distribution (d).

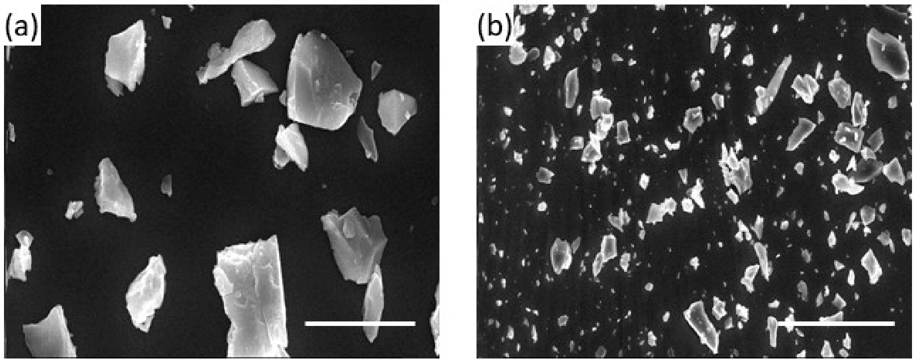

Figure S2. SEM images of 2D α-Ge (a) Scale bar of 2 μm and (b) Scale bar of 10 μm.

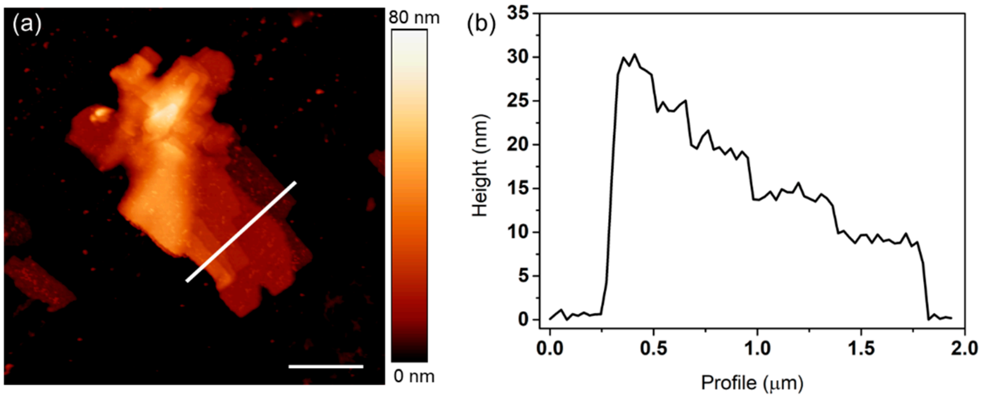

Figure S3. (a) AFM image of a typical 2D α-Ge. Scale bar of 1 μm. (b) Height profile along the white line in (a).

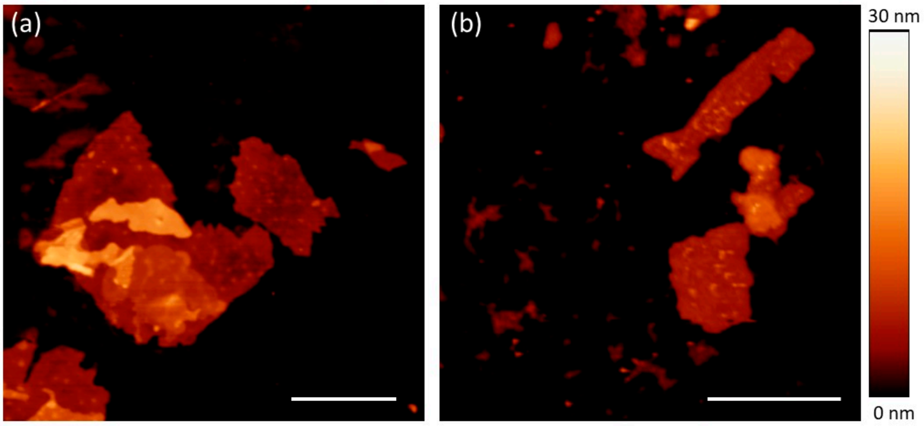

Figure S4. AFM topographic images of characteristic 2D α-Ge. Scale bar of 1 μm.

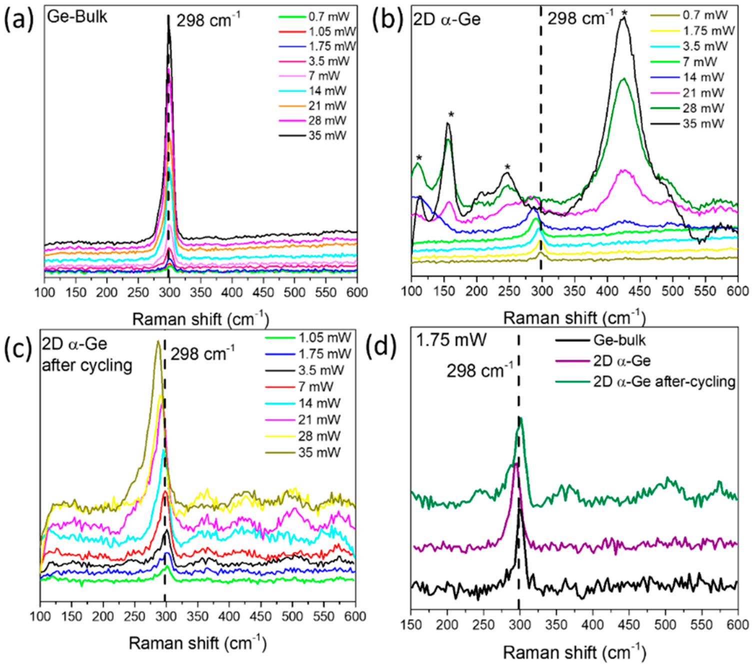

Figure S5. Raman spectra with different power for (a) Ge bulk (b) 2D α-Ge before cycling (c) 2D-α-Ge after cycling and (d) Raman spectra comparative with laser power of 1.75 mW.

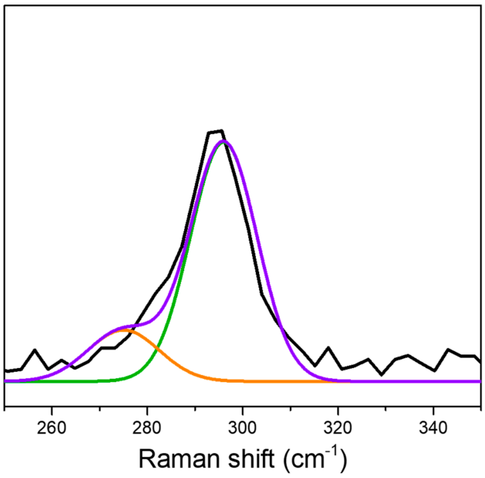

Figure S6. Raman Spectroscopy for 2D α-Ge before cycling at laser power of 3.50 mW.

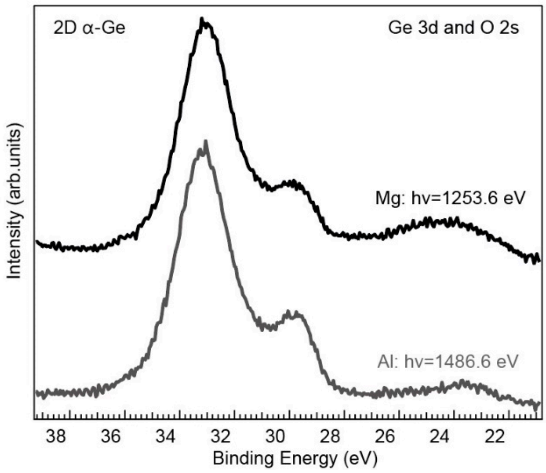

Figure S7. XPS spectra of the Ge 3d and O 2s region obtained with different photon energies. Top: 1253.6 eV (Mg Kα), bottom: 1486.6 eV (Al Kα). The relative intensity difference of the signal from elemental Ge (at 29.7 eV BE) is used for thickness calibration.

Section S3: Electrochemical characterization

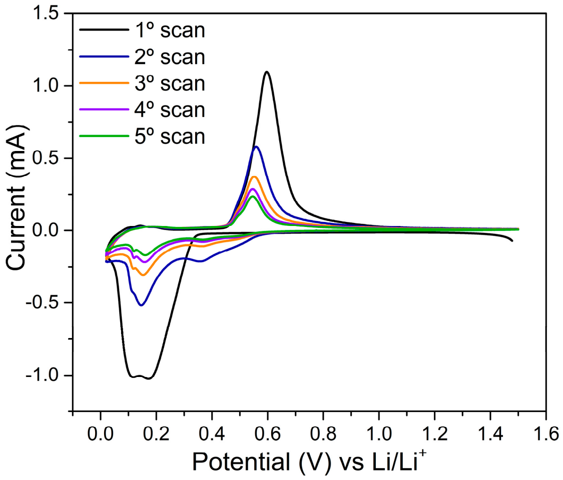

Figure S8. Cyclic voltammetry for 2D α-Ge 1 mg cm^{-2} first five cycles.

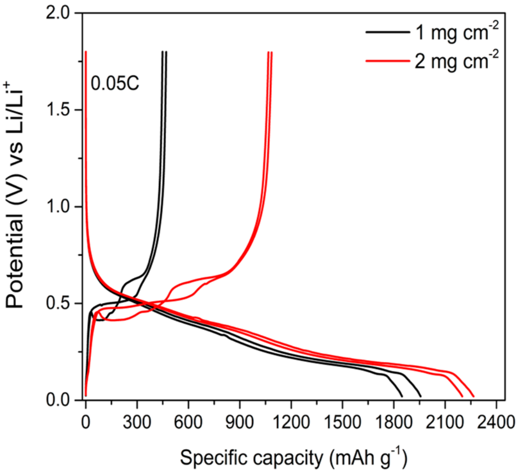

Figure S9. Specific capacity (mAh g^{-1}) of 2D α-Ge for 1 and 2 mg cm^{-2} two first cycles at 0.05C.

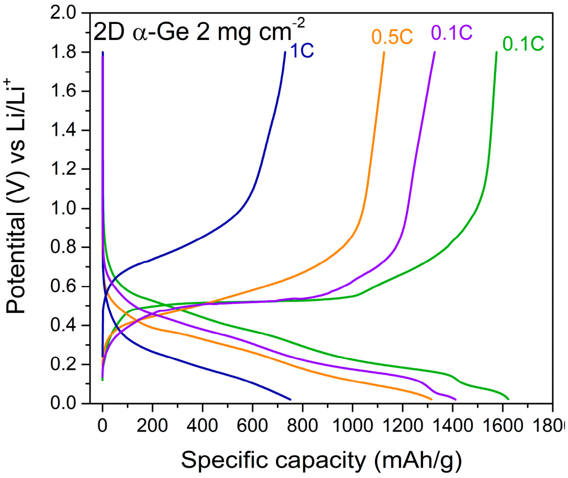

Figure S10. Specific capacity (mAh g^{-1}) of 2D α-Ge 2 mg cm^{-2} different scan rates.

Table S1. Specific capacity (mAh g^{-1}) at different scan rates.

Different scan rates	0.1C (initial)	0.5C	1C	0.1C (after 30 cycles)
2D α-Ge 1 mg cm^{-2}	1630	1396	729	1540
2D α-Ge 2 mg cm^{-2}	1630	1300	629	1413

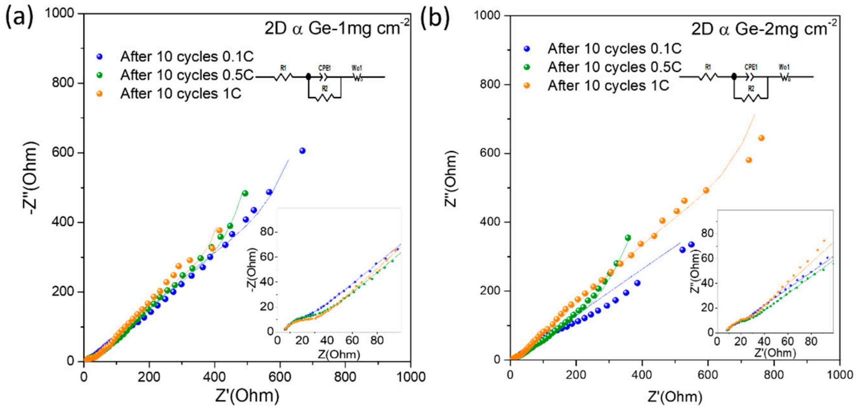

Figure S11. (a) Nyquist plot of 2D α-Ge 1 mg cm^{-2} different scan rates. (b) Nyquist plot of 2D α-Ge 2 mg cm^{-2} different scan rates.

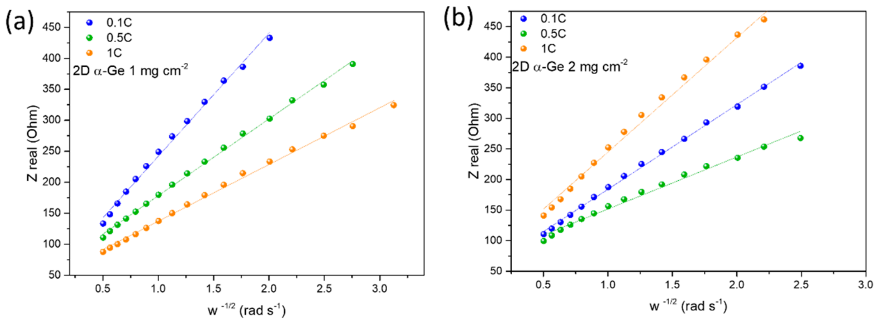

Figure S12. (a) Z real as function of $w^{-1/2}$ for 2D α-Ge 1 mg cm^{-2} and (b) 2D α-Ge 2 mg cm^{-2} at different scan rates.

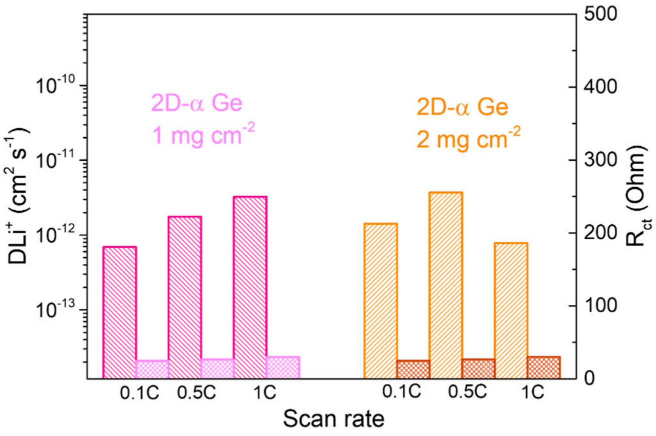

Figure S13. Li^+ diffusion coefficients (DLi^+) (filled columns) and R_{ct} (dashed columns) at different constant scan rate for 2D α-Ge 1 mg cm^{-2} semi-cell (pink bars), 2D α-Ge 2 mg cm^{-2} semi-cell (orange bars).

Table S2. Electrochemical impedance spectroscopy fitting parameters to R_s ($CPE_1//R_{ct}$) W_o

2D α-Ge 1mg cm^{-2} (Constant scan rate 0.5C)	Rs (Ω)	C(F)	n	Rct(Ω)	Wo-R	Wo-T	Wo-P	DLi$^+$ (cm^2 s^{-1})
After 10 cycles	7.8	$1.9\cdot10^{-4}$	0.71	50.5	15402	123	0.65	$1.02\cdot10^{-13}$
After 100 cycles	7.8	$1.8\cdot10^{-4}$	0.71	59.2	16626	129	0.68	$8.50\cdot10^{-14}$
After 200 cycles	6.1	$1.5\cdot10^{-4}$	0.60	43.8	1014	54	0.48	$1.50\cdot10^{-12}$
After 400 cycles	6.8	$2.0\cdot10^{-4}$	0.53	94.8	1222	68	0.47	$2.60\cdot10^{-12}$
2D α-Ge 1mg cm^{-2} (Different scan rate)	Rs (Ω)	C(F)	n	Rct(Ω)	Wo-R	Wo-T	Wo-P	DLi$^+$ (cm^2 s^{-1})
After 10 cycles 0.1C	3.9	$2.7\cdot10^{-4}$	0.78	9.96	1746	59.8	0.44	$6.90\cdot10^{-13}$
After 10 cycles 0.5C	4.6	$2.6\cdot10^{-4}$	0.72	20.0	1278	59.5	0.45	$1.80\cdot10^{-12}$
After 10 cycles 1C	4.5	$3.1\cdot10^{-4}$	0.68	21.0	1180	71.7	0.47	$3.20\cdot10^{-12}$
2D α-Ge 2mg cm^{-2} (Different scan rate)	Rs (Ω)	C(F)	n	Rct(Ω)	Wo-R	Wo-T	Wo-P	DLi$^+$ (cm^2 s^{-1})
After 10 cycles 0.1C	5.10	$2.08\cdot10^{-5}$	1.25	1.94	2472	696	0.38	$1.40\cdot10^{-12}$
After 10 cycles 0.5C	5.84	$2.04\cdot10^{-4}$	0.83	8.56	717	41.6	0.39	$3.80\cdot10^{-12}$
After 10 cycles 1C	6.53	$1.41\cdot10^{-4}$	0.84	8.52	2166	73.5	0.45	$7.80\cdot10^{-12}$

Section S4: Physicochemical characterization after cycling process.

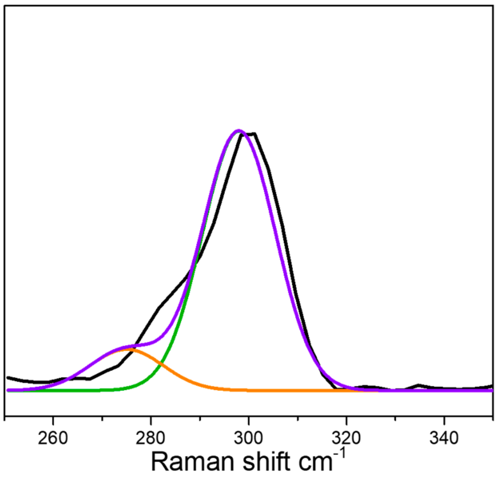

Figure S14. Raman Spectroscopy for 2D α-Ge after cycling at laser power of 3.50 mW.

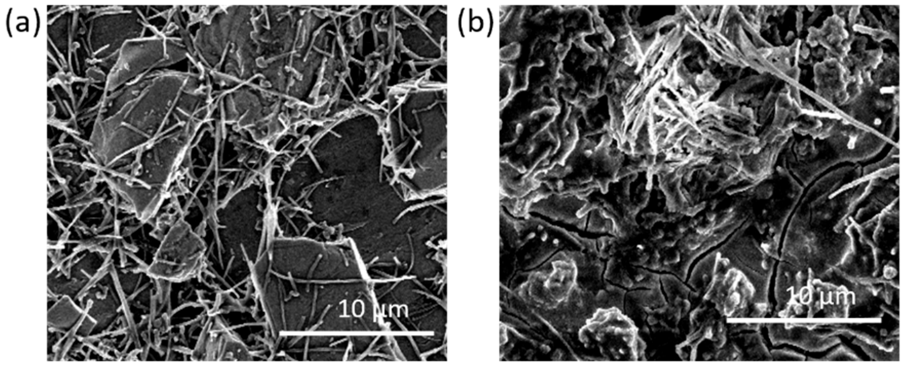

Figure S15. SEM images of 2D α-Ge anodes (a) before cycling (b) after cycling

References

1. Schmeisser, D.; Schnell, R.D.; Bogen, A.; Himpsel, F.J.; Rieger, D.; Landgren, G.; Morar, J.F. Surface oxidation states of germanium. *Surf. Sci. 172*, 455.
